# Association of Rare Recurrent Copy Number Variants With Congenital Heart Defects Based on Next-Generation Sequencing Data From Family Trios

**DOI:** 10.3389/fgene.2019.00819

**Published:** 2019-09-10

**Authors:** Yichuan Liu, Xiao Chang, Joseph Glessner, Huiqi Qu, Lifeng Tian, Dong Li, Kenny Nguyen, Patrick M. A. Sleiman, Hakon Hakonarson

**Affiliations:** ^1^Center for Applied Genomics, Children’s Hospital of Philadelphia, Philadelphia, PA, United States; ^2^Division of Human Genetics, Department of Pediatrics, The Perelman School of Medicine, University of Pennsylvania, Philadelphia, PA, United States; ^3^Department of Human Genetics, Children’s Hospital of Philadelphia, Philadelphia, PA, United States

**Keywords:** copy number variants, next-generation sequencing, congenital heart defects, large trios study, genomics

## Abstract

Congenital heart defects (CHDs) are a common birth defect, affecting approximately 1% of newborn children in the United States. As previously reported, a significant number of CHDs are potentially attributed to altered copy number variants (CNVs). However, as many genomic variants are rare, a large-scale CNV triad study is necessary to characterize the genetic architecture of CHD. We used whole-exome sequencing (WES) data generated by the Pediatric Cardiac Genomics Consortium (PCGC), including a discovery dataset of 2,103 individuals from 760 nuclear family trios and an independent replication set of 4,808 individuals from 1,712 trios. The candidate targets uncovered were further validated through different platforms, including the Omni single-nucleotide polymorphism (SNP) array chip in 1,860 individuals and the whole-genome sequencing (WGS) data in 33 trios. The genes harboring CNVs of interest were then investigated for expression alternations based on cardiac tissue RNA-Seq data. We identified multiple CNVs in the WES data that associated with specific sub-phenotypes of CHD in approximately 2,400 families, including 98 *de novo* CNV regions. We identified five CNV loci harboring *LIMS1*, *GCC2*, *RANBP2*, *TTC3*, and *MAP3K7CL*, respectively, where those genes are highly expressed in human heart and/or mouse embryo heart at 15 days. Five novel CNV loci were uncovered, demonstrating altered expression of the respective candidate genes involved. To our knowledge, this is the largest trio-based WES study of CHD and, in addition to uncovering novel CHD targets, presents an extensive resource with the potential to provide important insights to the architecture and impact of CNVs in CHD.

## Introduction

Congenital heart defect (CHD) is the most common class of major congenital anomalies in humans and a major source of morbidity and pediatric mortality around the world (van der Linde et al., 2011; van der Bom et al., 2011). The incidence estimates range from 4 to 10 in 1,000 live births (Hoffman and Kaplan, 2002). Genome-wide rare copy number variants (CNVs) with a minor allele frequency (MAF) < 1% are now recognized as an important contributor to CHD (Silversides et al., 2012; Soemedi et al., 2012; Greenway et al., 2009; Fakhro et al., 2011). Burden of large CNVs has been observed across CHD subtypes. The canonical 3-Mb 22q11.2 deletion has been observed as the most common recurrent *de novo* CNV associated with syndromic CHD. Recurrent *de novo* CNVs in patients with CHD reported in multiple studies also occur at chromosomes 1q21.1, 3p25.1, 7q11.13, 8p23.1, 11q24–25, and 16p13.11. Using exome paired with dense arrays, causal genes in these intervals have been identified, including *ELN* (Williams syndrome), *RAI1* (Smith–Magenis syndrome), *TBX1* (22q11 deletion), *GATA4* (8p23.1 deletion), *GJA5* (1q21.1 duplication), and *NKX2.5* (5q35.1 deletion). Recently, several large-scale studies have been conducted to explore the role of CNVs in CHD (Sifrim et al., 2016; Homsy et al., 2015; Glessner et al., 2014). Strikingly, recurrent *de novo* CNVs at 15q11.2 encompassing *CYFIP1*, *NIPA1*, and *NIPA2* were identified. Genes that interact with established CHD proteins NKX2-5 and GATA4 had singular *de novo* CNVs encompassing *DUSP1*, *JUN*, *JUP*, *MED15*, *MED9*, *PTPRE SREBF1*, *TOP2A*, and *ZEB2*. However, due to limited sample sizes, platform limitations, and inadequate control sets, significant potential to identify new CHD-related genes exists, especially for *de novo* genomic CHD CNVs. In this study, we used the largest trio dataset available for CHD to search for rare CNVs associated with CHD. The dataset included a discovery set of 2,103 individuals from 760 nuclear family trios, all whole exome sequenced (WES), and a mutually exclusive and independent replication set of 4,808 individuals from 1,712 trios with WES data. Other independent replication datasets available included an Omni single-nucleotide polymorphism (SNP) array dataset of 1,860 individuals (922 cases and 938 controls), and a whole-genome sequencing (WGS) dataset of 99 individuals from 33 trios; all individuals in the replication sets are mutually exclusive from the discovery set. The CNV target genes were further validated based on a differential gene expression test in 55 cardiac biopsy samples from 55 CHD patients from the replication dataset. As CHD is associated with other diseases, including developmental delays, we also consider other disease-associated CNVs beside *de novo* CNVs. Collectively, we uncovered five CNV loci that associated with CHD. These loci corresponded to five genes, *LIMS1*, *GCC2*, *RANBP2*, *TTC3*, and *MAP3K7CL*, all of which were altered in expression due to the presence of CNVs we uncovered.

## Materials and Methods

### Patient Cohorts

The sequence data and phenotypes were downloaded from the Database of Genotypes and Phenotypes (dbGaP) (accession phs001194.v2.p2 and phs001194.v2.p2.c1) released by the Pediatric Cardiac Genomics Consortium (PCGC). CHD probands and parents were recruited into the CHD Genes Study of the PCGC (CHD genes: ClinicalTrials.gov identifier NCT01196182) as previously described (Pediatric Cardiac Genomics C et al., 2013), using protocols approved by the institutional review boards (IRBs) of each institution. The CHD trios selected for this study had no history of CHD in first-degree relatives, for example, parents and direct siblings. CHD diagnoses were obtained from echocardiograms, catheterization, and operative reports; extra-cardiac findings were extracted from medical records and included dysmorphic features, major anomalies, noncardiac medical problems, and deficiencies in growth or developmental delay. The etiologies for CHD were unknown; patients with previously identified cytogenetic anomalies or pathogenic CNVs identified through routine clinical evaluation were excluded. Whole-blood samples were collected, and genomic DNA was extracted. Cardiac tissue samples were collected during surgery for RNA extraction.

Patients recruited were divided into several datasets (**Table 1**), including a discovery dataset for WES (2,103 individuals from 760 nuclear family trios, including 652 families with at least one parent/sibling); a replication dataset for WES (4,808 individuals from 1,712 family trios, including 1,570 families with at least one parent/sibling); SNP array replication dataset (1,860 unrelated individuals); and WGS replication set (99 individuals from 33 family trios), and individuals in all replication datasets are mutually exclusive to the discovery set. An RNA-sequencing dataset was applied as the functional validation dataset for gene expression alternations (55 cardiac tissue samples). All the controls in these datasets are the first-degree relatives (parents or siblings) of the CHD patients except for the SNP array replication set. The discovery set is exclusive to the replication and validation datasets.

**Table 1:** Summary of the patient cohorts and genomic technologies employed in this study.

Dataset	Platform	Size
Discovery	WES	759 probands and 1,346 controls from 760 trios
Replication A	WES	1,699 probands and 3,109 controls from 1,712 trios
Replication B	Array CHIP	1,860 individuals, 922 proband
Replication C	WGS	33 proband and 66 controls from 33 trios
Validation	RNA-Seq	55 cardiac tissues from 55 CHD probands

WES, whole-exome sequencing; WGS, whole-genome sequencing.

### Generating CNV Targets

**Figure 1:**
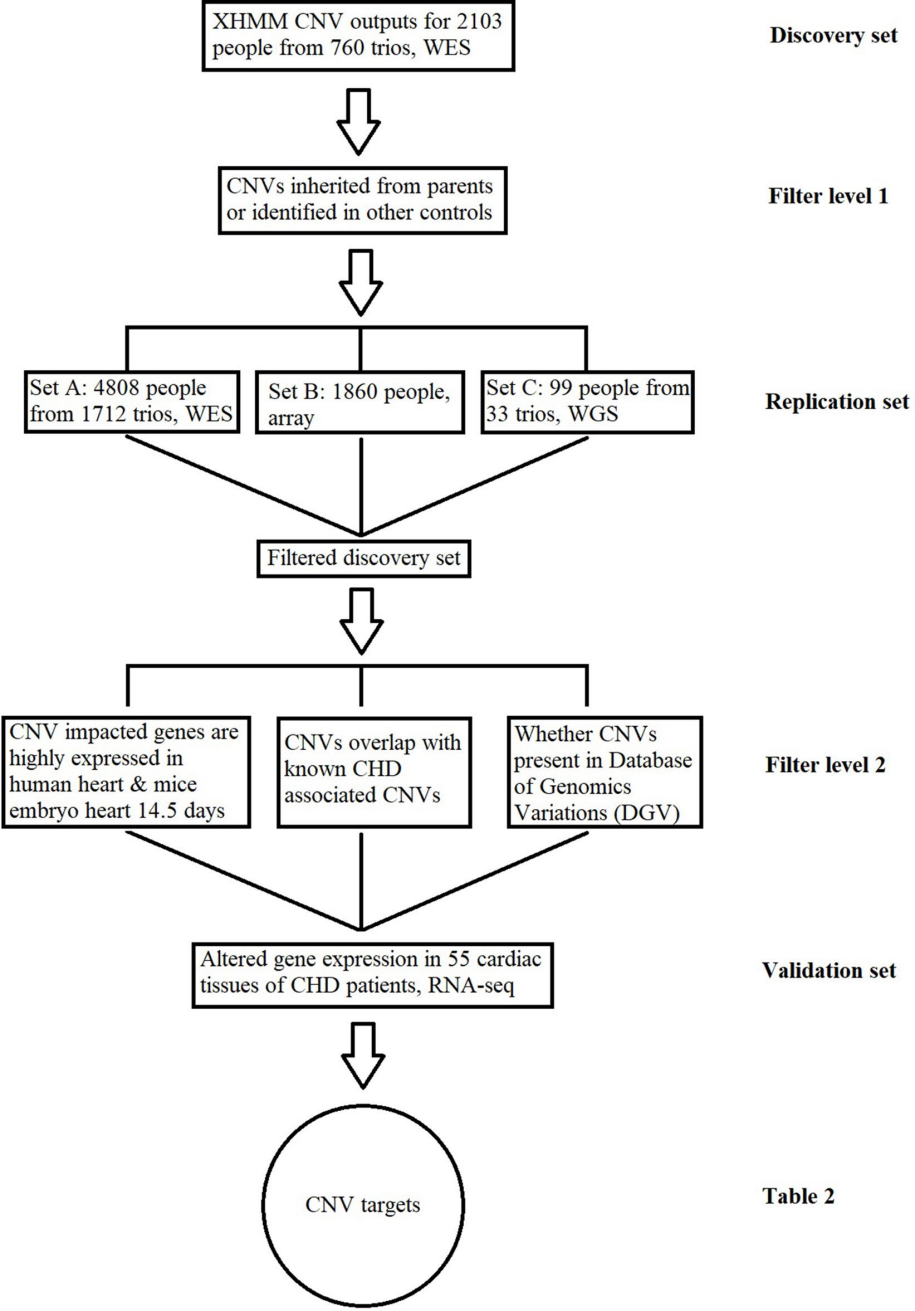
Copy number variant (CNV) identification and functional validation pipeline. This figure describes the conceptual pipeline for CNV target selection based on large sequencing/chip data of different platforms. The CNVs for the whole-exome sequencing (WES) data in the discovery set from 760 trios were detected based on the XHMM pipeline. Any inherited CNVs or CNVs identified in independent healthy controls were filtered at the first level; the remaining CNVs were further replicated in three exclusive datasets at different platforms and checked through previously reported results for further confirmation; the expressions for CNV-impacted genes were compared between the patients with/without the CNV based on RNA-Seq of cardiac tissues. CNVs associated with altered gene expression are listed in **Table 2** as the targets for CHD.

In order to identify causal CNVs in the CHD samples, we set up a discovery pipeline based on several criteria (**Figure 1**). Methods applied to identify CNVs in different platforms were described in the following paragraphs. In general, for the CNVs found in the discovery set, we required that target CNVs were not inherited nor present in any independent family controls. The resulting CNVs were subsequently checked for individual replication in the independent datasets, and the CNVs were removed if they were found in any of the replication control datasets. In other words, the filtered discovery set (**Table S1**) required the CNVs be *de novo* while not identified in any controls and at least replicated in one set. After this step, additional filters were applied based on the prior biological knowledge of CHD, including the following: 1) the CNV corresponding genes are highly expressed in human or mouse embryo during development at 14.5 days (Zaidi et al., 2013); 2) the CNV is not physically overlapped with any previously reported CHD-associated/causal CNVs based on genomic locus (Glessner et al., 2014); 3) the CNV has not been previously reported as common CNVs in healthy individuals through the Database of Genomic Variations (DGV) (MacDonald et al., 2014). CNVs in **Table S1** that fit at least one of these requirements were selected as a higher confidence target list (**Table S2**).

### Statistical Analysis

CNV calling software with different algorithms has varied reproducibility for NGS data. A previous study showed that CNV callers with read depth algorithm (XHMM, ExomeDepth, and CODEX) are beyond performance compared with others, especially in rare CNV detection (D O’Fallon et al., 2018). XHMM is less sensitive than ExomeDepth and CODEX, but much more precise than them (93% vs 54% and 72%) (Sadedin et al., 2018). In this study, CNV calling was performed using the standard XHMM pipeline consisting of six steps (Fromer et al., 2012). 1) The depth of coverage for all targets and all CHD samples used for subtype comparison was performed using GATK. 2) Target regions with extreme GC content (< 10% or >90%), and low-complexity regions were filtered out from further analysis. 3) Principal component analysis (PCA) normalization of read depth was performed for all samples to remove inherent biases in sample preparation and sequencing. 4) Samples with extreme variability in normalized read depth were removed. 5) *Per*-sample CNV detection with a hidden Markov model (HMM) was performed. 6) Quality metrics were assigned to all samples for discovered CNVs. One thousand eight hundred sixty individuals were genotyped on the Omni1M and Omni2.5M arrays. CNV calls were generated by the HMM-based software PennCNV as we described before (Wang et al., 2008). WGS CNVs were called by standard pipelines of BreakDancer (Fan et al., 2014), and we required at least 50% of reads to support the CNV identification. For RNA-Seq data of the corresponding CNV protein coding genes, we compared the estimated expression levels that were measured as fragments *per* kilobase of transcript *per* million (FPKM) in CHD individuals with/without CNVs by Cuffdiff and Cuffnorm (Trapnell et al., 2010).

## Results

### CNV Targets

Due to the high prevalence of CNVs across human genomes, the most significant difficulty of CNV analysis is to identify and remove the CNVs that are unrelated to a disease, such as CHD (Stankiewicz and Lupski, 2010). This study used three critical steps to remove CNV hits irrelevant to CHD. A recent study based on a large WES dataset showed that CNVs inherited from unaffected parents were usually benign in comparison with CNVs that were *de novo* and unique to the proband (Sifrim et al., 2016). Therefore, at the first step, we removed all the CNV hits which either were inherited or were found to overlap with other independent controls of our dataset before we check the replication dataset. Considering the limitation of this study that different genomic platforms with different sensitivities and preferential detection of different types of CNVs were used for CNV calling, at the second step, we check the CNV hits through three mutually exclusive independent replication datasets from different genomic platforms, including WES, SNP array chips, and WGS, in order to determine support for CNV causality of CHD. We identified 122 CNV regions which are supported by at least one replication dataset (**Table S1**). For biological relevance, we screened through previously published databases and reduced the candidate CNV targets group to 39 (**Table S2**). In addition, a list of 98 *de novo* CNVs never reported in any publication before based on the DGV database, including 35 recurrent CNVs, was generated for the CHD probands (**Table S3**).

### Functional Validation Based on Cardiac Tissues

**Figure 2:**
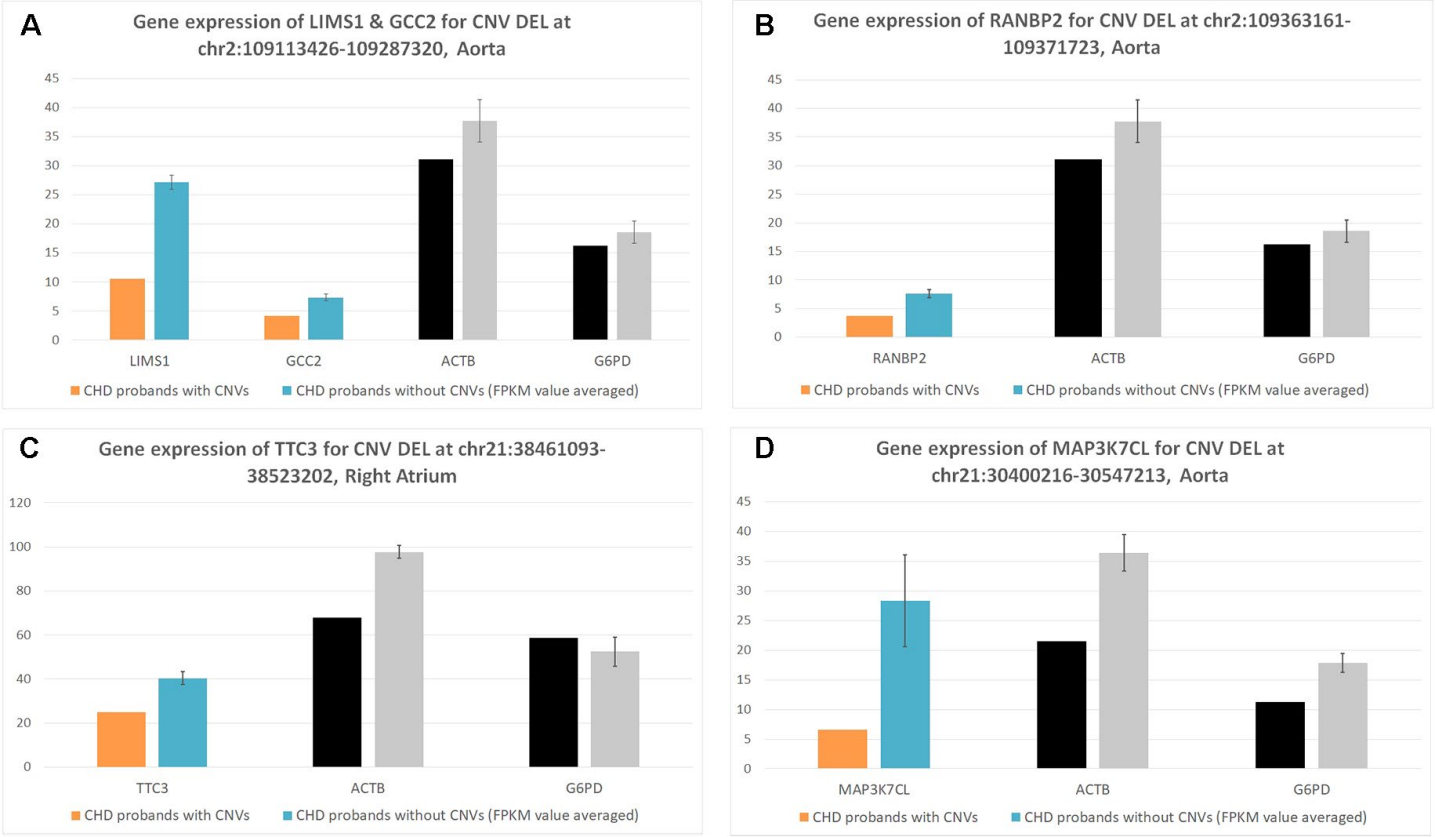
Corresponding gene expression for target copy number variants (CNVs). These figures represent the corresponding CNV gene expression [*Y* axis of normalized fragments *per* kilobase of transcript *per* million (FPKM) value ranged from 0 to 100] for CHD probands who contain the CNV (case) versus CHD probands who do not contain the CNV (controls). Each bar is a CHD proband, and each cluster of bars indicates a certain gene. Housekeeping genes *G6PD* and *ACTB* were selected as the baseline control to normalize gene expression levels. **(A)** Deletion for LIMS1 and GCC2 genes at chr2:109113426-109287329 in aorta. **(B)** Deletion for RANBP2 gene at chr2:109363161-109371723 in aorta. **(C)** Deletion for TTC3 gene at chr21:38461093-38523202 in right atrium. **(D)** Deletion for MAP3K7CL AT chr21:30400216-30547213 in aorta.

As DNA structural variations, such as CNVs, can lead to expression alternations for corresponding genes by copy number change or duplication/deletion of certain exons or regulatory elements, we examined the corresponding gene expression alternations between probands harboring the target CNVs and probands that do not. Due to the difficulty in collecting healthy people’s cardiac tissues, the validation was done in probands who had cardiac biopsy available through surgery. Four CNV regions were validated through this process (**Tables 2** and **3**, **Figure 2**).

**Table 2:** Identification of CNV targets in CHD.

CNV	Discovery set				WES replication A	Array (replication B)	WGS (replication C)
	Type	Occurrences of CHD	DGV checking	Corresponding gene	CNV regions	CNV regions	CNV regions
chr2:109363161-109371723	DEL	4	*De novo* deletion, duplication related to evolution, and diversity of CNV in the great ape lineage, 10 CHD probands contain the duplications	RANBP2	1-02327 (chr2:109365376-109389041, DUP), 1-03696 (chr2:109369454-109389502, DUP); 1-02846^*^ (chr2:108604612-110350712, DEL); 1-07110 (chr2:109371361-110350712, DUP); 1-00391 (chr2:109113426-109371540, DEL)	NA	NA
chr2:109113426-109287320	DEL/DUP	3	The CNV region associated with developmental delays	*LIMS1*, *GCC2*	1-02846^*^ (chr2:108604612-110350712, DEL); 1-04724^*^ (chr2:109124002-109124101, DUP), 1-05788 (chr2:109113426-109287320, DUP); 1-00391 (chr2:109113426-109371540, DEL)	PCGC0043294 (chr2:109173930-109301074, DEL)	NA
chr21:38461093-38523202	DEL	1	Deletion is associated with developmental delays, and duplication is common in the CNV region	TTC3	1-03431 (chr21:38495256-38501383, DEL); 1-01155 (chr21:38459558-38522474, DEL); 1-03555 (chr21:38461093-38523202, DEL); 1-03784^*^ (chr21:38461093-38467747, DEL); 1-00180 and 1-04922 (chr21:38460105-38468962, DEL); 1-02727^*^ (chr21:38462510-38505074, DEL)	NA	NA
chr21:30400216-30547213	DEL	1	*De novo* CNVs (not in DGV)	MAP3K7CL	1-01557^*^ (chr21:30402916-30414871, DEL)	PCGC0042591 (chr21:30444607-30656199, DEL)	1-02231* (chr21:30545987-30546715, DEL); 1-05672 (chr21:30426625-30427238, DEL)

CNV, copy number variant; CHD, congenital heart defect; DGV, Database of Genomic Variations; WES, whole-exome sequencing; WGS, whole-genome sequencing; NA, not applicable.

*These CHD patients have cardiac biopsy samples.

**Table 3:** RNA-Seq validation for target CNVs.

Gene ID	CHD biological relevance	Previously reported CHD CNVs	Disease associated	RNA-Seq tissue	FPKM proband with CNV	FPKM proband without CNV	*p* value
*LIMS1*	*LIMS1* is highly expressed in mouse at 14.5 days and human heart	PMID: 25205790, 22969434	Conotruncal heart malformations	Aorta	10.58	27.18	0.01475
				Left ventricle	11.82	14.73	0.5798
*GCC2*	NA	PMID: 25205790	NA	Aorta	4.20	7.34	0.1288
				Left ventricle	6.96	10.88	0.3288
*RANBP2*	*RANBP2* is highly expressed in mouse at 14.5 days and human heart	PMID: 22155005	Encephalopathy	Aorta	3.77	7.62	0.0596
*TTC3*	*TTC* is highly expressed in mouse at 14.5 days and human heart tissue	PMID: 25205790, 22912673	Down syndrome	Right ventricle	25.51	33.40	0.5017
				Right atrium	24.85	40.45	0.01965
*MAP3K7CL*	Overexpressed in the coronary artery and aorta	NA	Coronary artery disease	Aorta	6.55	28.32	0.01435
				Atrial septum	6.43	2.71	0.2783

CHD, congenital heart defect; CNV, copy number variant; FPKM, fragments per kilobase of transcript per million; NA, not applicable.

We uncovered five CNV targets as shown in **Table 2**. A CNV residing at chr21:30400216-30547213 is a *de novo* deletion identified in a single CHD proband (1–01968) and was replicated in multiple dataset including the WES, SNP array, and WGS datasets as shown in **Table 2**. The corresponding gene *MAP3K7CL* is overexpressed in arteries, including coronary arteries and the aorta, and associated with coronary artery disease (Miller et al., 2016), and a recent genome-wide association study (GWAS) for heart failure suggests it is the top risk gene for coronary artery disease (Aragam et al., 2018). Aorta biopsy was selected, and the expression of *MAP3K7CL* was found to be reduced significantly by the CNV (*p* value = 0.014). In contrast, in atrial septum biopsy samples, the expression of *MAP3K7CL* increased about threefold in CHD patients compared to controls (**Figure S1**). These findings may imply tissue specificity for the CNV’s impact on gene expression and association with tissue-specific CHDs, which warrants further study.

The deletion CNV at chr2:109113426-109287320, corresponding to the *LIMS1* and *GCC2* genes, was detected in three CHD probands in the discovery dataset and replicated in two independent CHD probands in the WES replication dataset (permutation test *p* = 5.18 × 10^−3^). This region has been reported as a CHD-associated region (Glessner et al., 2014), and based on the Database of Genomic Variants (DGV), the region has been reported as a locus for developmental delay (Coe et al., 2014). *LIMS1* is highly expressed in mice at day 14.5 and in human heart (Zaidi et al., 2013) and is associated with conotruncal heart malformations in multiple studies (Saraclar et al., 1996). Both *LIMS1* and *GCC2* show significant reduction of expression in the aorta tissue in CHD probands (subjects 1-02486 and 1-04724) who have this CNV, in comparison with probands (*n* = 7) who do not harbor this CNV (*p* value = 0.015 and 0.12).

The deletion CNV at chr2:109363161-109371723, corresponding to the gene *RANBP2*, existed in four CHD probands from the discovery dataset and was replicated in two independent individuals from the replication WES dataset without any occurrences in controls (permutation test *p* = 2.13 × 10^−3^). The deletion CNV is a *de novo* variation, and the *RANBP2* gene is highly expressed in mouse at day 14.5 and in the human heart (Zaidi et al., 2013; Kim et al., 2012). Previous studies showed that the gene is the target for small ubiquitin-related modifier (SUMO) conjugation and could lead to CHDs and cardiac dysfunction in murine hearts (Kim et al., 2012; Sakin et al., 2015). The aorta tissue biopsy was collected for the CHD proband, 1-02846, who has the target CNV deletion and seven other CHD probands who do not have the CNV. The expression of *RANBP2* was significantly reduced (*p* value = 0.05) while two housekeeping genes remained stable, indicating the expression alternations in *RANBP2* as a consequence of the targeted CNV.

The deletion CNV at chr21:38461093-38523202, corresponding to the gene *TTC3*, existed in a single CHD proband, 1-04198, in the discovery dataset and was replicated in seven independent individuals from the WES replication dataset without any occurrences in controls. The deletion CNV overlaps with CNVs previously reported in CHD studies (Glessner et al., 2014) and associated with developmental delay (Coe et al., 2014). *TTC3* is highly expressed in mouse at 14.5 days and in human heart tissue (Zaidi et al., 2013); a previous study showed that the gene correlates with cardiac defects in Down syndrome patients (Ripoll et al., 2012), and *TTC3* is a novel candidate gene based on array comparative genomic hybridization (aCGH) for 316 nonsyndromic CHD children (Sanchez-Castro et al., 2016). The right ventricle and right atrium tissue biopsies were collected for CHD probands, 1-02727 and 1-03784, who have the target CNV deletions as well as for six other CHD probands who do not harbor the CNV. The expression of *TTC3* was significantly reduced in both tissues while two housekeeping genes remained stable, indicating that the expression alternation is attributed to the targeted CNV. Mechanistic reasoning regarding the role of *TTC3* in CHD warrants reconciliation with the observed increased gene dosage in Down syndrome and the deletion observed in our study.

## Discussion

### New CNV Loci Identified in This Study

CHD is the most common birth defect, affecting approximately 1% of newborns, and research has shown that genomic variations contribute significantly to CHD. Recent studies from large CHD populations demonstrate that CHD is associated with other birth conditions such as developmental delay (Homsy et al., 2015). CNVs have been reported previously in association with CHD where rare recurrent *de novo* CNVs are enriched in CHD cases (Glessner et al., 2014). Compared with the previous study where 65 *de novo* CNVs identified in CHD cases, our study identified 122 CNVs which are supported by at least one replication dataset, but not seen in controls We also performed a family-based data analysis from over 2,500 families with CHD probands to explore the impact of non-inherited CNVs in the pathogenesis of CHD. We used data from different platforms including WES, SNP arrays, WGS, and RNA sequencing. Four layers of filters based on independent replication sets were applied to remove CNVs unrelated to CHD, in order to reduce false-positive hits.

Beside the independent replications, we also performed evaluation based on various biological studies from existing knowledge bases, and functional studies at mRNA levels were used to lend further pathogenic support to variants of interest. We required target genes to be highly expressed in human heart, blood vessel tissues, or mouse embryo heart, an approach previously established in relation with CHD-associated CNVs (Glessner et al., 2014; Zaidi et al., 2013). In the filtered list of 39 CNVs (**Table S2**), 29 are *novel* CNVs while 10 CNVs overlapped with developmental delay regions. These results are consistent with previous reports which demonstrated association between CHD and developmental delay, as many of our targeted CNVs are associated with developmental delays and other developmental disease loci, which brings more confidence to the results.

The targeted CNVs were further validated through cardiac biopsies obtained from individuals harboring the targeted CNVs. Indeed, the corresponding genes impacted by targeted CNVs showed significantly altered gene expression in comparison with individuals without targeted CNVs, while the housekeeping genes are stable (**Table 3**, **Figure 2**). Ideally, affected probands with CNVs would be compared to unaffected controls; however, due to the natural difficulties in obtaining healthy cardiac tissues, we had to compare the gene expressions among CHD probands. These results demonstrate that the targeted genomic CNV does impact gene expression and are potential pathogenic culprits in CHD.

### *RANBP2*: A Link Between Neurodevelopmental Disorders (NDDs) and CHD

Among recurrent *de novo* CNV examples, a deletion at chr2:109363161-109371723, which deletes exons 8 to 17 of the *RANBP2* gene, was observed recurrently in four independent probands, but not in any of the control samples including their parents or siblings. *RANBP2* is a small guanosine-5′-triphosphate (GTP)-binding protein of the RAS superfamily that is associated with the nuclear membrane and is thought to control a variety of cellular functions through its interactions with other proteins. The encoded protein directly interacts with the E2 enzyme *UBC9* and strongly enhances *SUMO1* transfer function. A previous study showed that both heterozygous and homozygous *SUMO1* knockout mice exhibit atrial septal defect (ASD)/ventricular septal defect (atrial septal defect) and suffer from high mortality rates; this was rescued by cardiac re-expression of the *SUMO1* transgene (Wang et al., 2011). Our results indicate that a large deletion of *RANBP2* results in the reduction of the *SUMO1* transfer function, which may explain the association with CHD in humans. The CNV region was further validated in two more CHD probands from an independent trio data. Aorta tissues were also obtained from one of the proband and from several CHD cases without this CNV deletion. As show in **Figure 2B**, the expression of *RANBP2* (blue bar) is significantly reduced in the CNV probands, while the housekeeping genes *G6PD* and *ACTB* remain stable in all subjects tested. Thus, it is exceedingly likely that the deletion is responsible for the gene expression reduction and could further impact the *SUMO1* transfer pathway in CHD cases with a high mortality rate.

NDD is commonly seen in CHD patients (Marino et al., 2012). Shared genetic etiology of NDD and CHD has been identified in genes involved in morphogenesis, chromatin modification, and transcriptional regulation (Homsy et al., 2015). As shown previously, RanBP2 works as a chaperone with the mitochondrial metallochaperone Cox11 (encoded by the cytochrome c oxidase copper chaperone *COX11* gene) and plays critical roles in the modulation of neuronal hexokinase type I (HKI), which is the pacemaker of glycolysis (Aslanukov et al., 2006). Cox11 inhibits HKI activity, and RanBP2 suppresses this inhibition. Haploinsufficiency of *RANBP2* in mice induces the downregulation of HKI and ATP levels selectively in the central nervous system (Aslanukov et al., 2006). Targeting at this mechanism, e.g., to activate HKI, may offer new opportunities to impact NDD in CHD patients.

### *LIMS1*: A Link Between Innate Immunity and CHD

Besides *RANBP2*, the other CNV locus at chromosome 2 containing the gene *LIMS1* may provide important insight into innate immunity and CHD, based on its significant change of gene expression and previous studies of its role in CHD (Glessner et al., 2014; Saraclar et al., 1996). As shown in this study, the CNV locus at chr2:109113426-109287320 was associated with a significantly decreased expression of *LIMS1*. In mouse models, a previous study has shown that the *LIMS1* protein forms a functional complex with thymosin β4 and integrin-linked kinase, which plays an important role in cardiomyocyte migration, survival, and repair (Bock-Marquette et al., 2004). In humans, *LIMS1* polymorphism has been identified in association with monocyte chemotactic protein-1 (MCP1) levels (Ahola-Olli et al., 2017). It is one of the key chemokines that regulate migration and infiltration of monocytes/macrophages (Deshmane et al., 2009), while the critical role of macrophages in neonatal heart regeneration has been demonstrated (Aurora et al., 2014). A previous study on rare CNV in congenital left-sided heart disease also identified a paternally inherited loss of *LIMS1* in a case with partially anomalous pulmonary venous return and ligation of patent ductus arteriosus (Hitz et al., 2012). In addition, considering the role of macrophages in resistance to virus infections, *LIMS1* might also represent a link of intrauterine virus infections and congenital heart disease.

### Conclusions

CNVs have been previously shown to contribute towards CHDs. However, limited sample sizes have prevented these studies from discovering recurrent CNVs in genes contributing towards CHD. To explore the impact of recurrent rare CNVs, we performed the largest family-based CHD structural variation genomic study to date, identifying targets we replicated using different technical platforms based on independent data, and the CNV target regions were further validated through CHD-related knowledge and cardiac tissue gene expression alternations. The new insights into CHD provided by this study offer new opportunities to clarify the development of CHD and its comorbidities.

This study has limitations. For CNVs’ impact on gene expression, we showed unadjusted *p* values in this paper, which lose significance after correction for multiple testing due to the difficulty in obtaining the statistical power. It is difficult to acquire healthy human heart tissues, and there are limited RNA-Seq data available in public databases such as dbGaP. We could only compare the CNV-impacted gene expression between CHD patients with target CNVs and CHD patients without target CNVs. The tendency of gene expression changes associated with the existence of target CNVs shown in this study warrants validation in future studies. As another limitation of our study, we had only accession to anonymous data of the CHD patient collection, without detailed phenotyping information about the clinical characteristics of CHD. We acknowledge the importance of a clinical investigation on genotype–phenotype analysis for the rare CNVs identified in our study, in the near future, working with the PCGC.

## Data Availability

Data can be accessed by the dbGaP accession phs001194.v2.p2 and phs001194.v2.p2.c1.

## Ethics Statement

The protocol was approved by the Institutional Review Boards of Boston Children’s Hospital, Brigham and Women’s Hospital, Great Ormond St. Hospital, Children’s Hospital of Los Angeles, Children’s Hospital of Philadelphia, Columbia University Medical Center, Icahn School of Medicine and Mt. Sinai, Rochester School of Medicine and Dentistry, Steven and Alexandra Cohen Children’s Medical Center of New York, and Yale School of Medicine. Written informed consent was obtained from each participating subject or their parent/guardian.

## Author Contributions

HH and PS conceived and supervised the project. YL, XC, HQ, and JG designed and implemented the methods. LT, DL, and KN contributed to data acquisition and analysis. YL, HQ, XC, JG, and HH wrote the manuscript. All authors approved the manuscript.

## Conflict of Interest Statement

The authors declare that the research was conducted in the absence of any commercial or financial relationships that could be construed as a potential conflict of interest.

## Supplementary Material

Figure S1 Corresponding gene expression for target copy number variants (CNVs) that do not have significant alternations. Similar to **Figure 2** except the target CNV regions in this plot do not have gene expression alteration significantly while housekeeping genes are stable. For pulmonary valve leaflets tissue, since *ACTB* is not expressed, *B2M* was selected as a housekeeping gene.

Table S1 CNV targets that not occurred in any health controls, and supported by at least one evidence from previous knowledge, biological supports or replication set.

Table S2 Selected CNV targets based on replication set, previous knowledge and biological supports.

Table S3 De novo and novel CNV targets identified in the discovery set.
