# Supplementary figures and images for: Association of Rare Recurrent Copy Number Variants With Congenital Heart Defects Based on Next-Generation Sequencing Data From Family Trios
Source: Front Genet. 2019 Sep 10;10:819. doi: 10.3389/fgene.2019.00819 (PMC6746959; doi:10.3389/fgene.2019.00819)

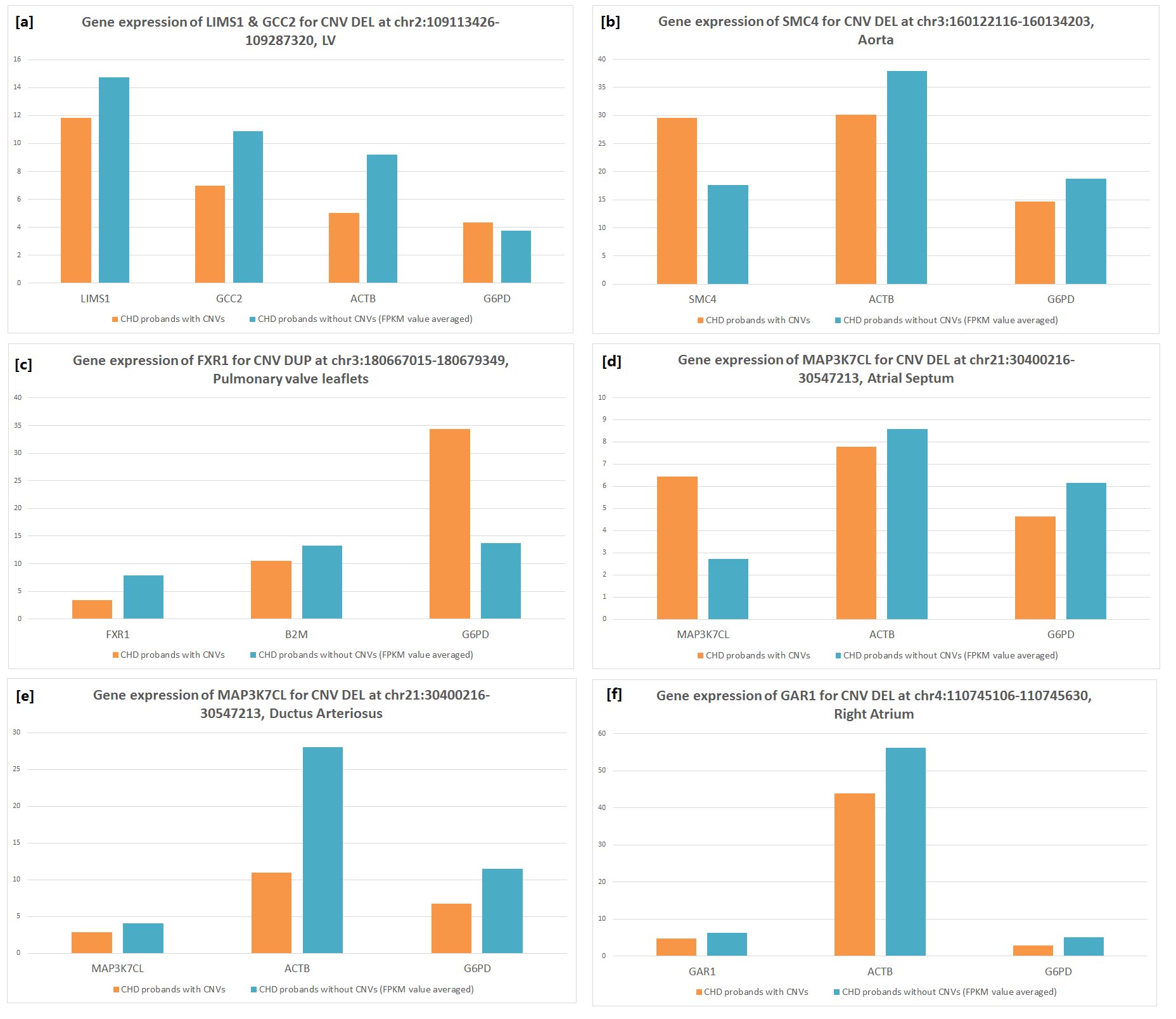

Supplement: Figure S1 — Corresponding gene expression for target copy number variants (CNVs) that do not have significant alternations. Similar to Figure 2 except the target CNV regions in this plot do not have gene expression alteration significantly while housekeeping genes are stable. For pulmonary valve leaflets tissue, since ACTB is not expressed, B2M was selected as a housekeeping gene. [file Image_1.png]
